# Genomic and transcriptomic insights into *Trichomonascus vanleenenianus*, a xylan-degrading yeast isolated from saproxylic insect larvae

**DOI:** 10.1186/s12864-026-12750-7

**Published:** 2026-03-21

**Authors:** Anita Boisramé, Frédéric Bigey, Martine Pradal, Cécile Neuvéglise

**Affiliations:** 1 SPO, Univ Montpellier, INRAE, Institut Agro, 34060 Montpellier, France; 2 AgroParisTech, Université Paris-Saclay, Palaiseau, France

**Keywords:** Genome sequence, CAZymes, Xylan degradation, Xylanase, Transcriptome

## Abstract

Studying microbial biodiversity offers enormous potential for improving the valorisation of plant biomass, particularly in conversion processes. Microorganisms, such as bacteria, fungi, yeasts and algae, play an essential role in degrading plant biomass and can be exploited to maximise the conversion of organic matter into valuable products. Of particular importance is the ability to use yeast strains that can degrade and utilise xylan. We sequenced and annotated the genome of a new strain of *Trichomonascus vanleenenianus* isolated from the gut of a saproxylic insect larva that exhibits this capacity. The genome of strain L1-24 (= CLIB 3782) was assembled into 116 nuclear scaffolds totaling 14.9 Mb. Comparative genomics analysis revealed that this species is phylogenetically closely related to the species *Blastobotrys mokoenaii*, *Blastobotrys malaysiensis* and *Blastobotrys illinoisensis*. Genome analysis revealed characteristics including a large number of spliceosomal introns (2,362 in the CDS of 1,706 genes and 176 in the UTRs) and the presence of all 5S rRNA genes arranged in tandem with tRNA genes. Protein prediction identified a total of 192 carbohydrate-active enzymes, many of which potentially act on plant cell wall polymers. Like the three other closely related species, *T. vanleenenianus* possesses the complete enzymatic repertoire necessary for the hydrolysis of xylan, including a key functional GH11 endoxylanase that is unique to these four species. Transcriptomic analysis in the presence of xylan revealed the induction of at least five xylan-degrading genes, including one coding for a protein belonging to the GH5_22 subfamily, as well as several transporters that could specialise in the uptake of xylooligosaccharides by yeast. Finally, monitoring xylan degradation and sugar uptake was performed using a fluorescent reporter protein under the control of the *XYL1* promoter.

**Supplementary Information:**

The online version contains supplementary material available at 10.1186/s12864-026-12750-7.

## Introduction

Adding value to plant biomass represents a major challenge, but also a major opportunity for a greener and more sustainable economy. Plant biomass, which encompasses organic matter derived from plants such as agricultural residues, forestry waste or energy crops, can be transformed into a variety of useful products, including energy, materials, chemicals and even bioplastics. This transformation is based on conversion processes that can be thermochemical, mechanical or biological, such as microbial conversion. However, for bioconversion, the complex structure of lignocellulose requires the feedstock to be pretreated using various chemical, physical and enzymatic methods to deconstruct the cell wall structure.

Lignocellulosic biomass is composed of two main polysaccharides: cellulose, a linear polymer of D-glucose units linked by ß-(1,4)-glycosidic bonds, and hemicellulose, a more heterogeneous branched polysaccharide, and of an aromatic polymer, lignin, which is mainly responsible for the recalcitrance of lignocellulose [1]. Hemicellulose comprises three main polymers: xylan, which consists of D-xylose units linked by ß-(1,4)-glycosidic bonds, substituted by α−1,2- or α−1,3-linked arabinosyl units and α−1,2-linked (methyl)-glucuronic acid moieties; glucomannan, a polymer of D-glucose and D-mannose units linked by ß-(1,4) glycosidic bonds, with branched D-galactose; or xyloglucan, a polymer of D-glucose units linked by ß-(1,4) glycosidic bonds, with branched D-xylose and D-galactose [2]. These hemicelluloses can be extensively acetylated with acetyl groups, which act as fermentation inhibitors that hinders bioconversion of lignocellulose. The composition of lignocellulosic biomass and the proportion of each component vary depending on the plant species and their classification, such as hardwoods, softwoods and grasses.

Microorganisms are currently used in biological pretreatment to break down lignocellulose. They modify or degrade lignocellulose extracellularly by secreting carbohydrate-active enzymes (CAZymes) and ligninolytic enzymes that depolymerise lignin [1]. Many bacteria, including *Clostridium spp.*, *Cellulomonas spp.*, *Bacillus spp.*, or *Streptomyces spp*., and several fungi such as *Phanerochaete chrysosporium*, *Trichoderma reesei* or *Aspergillus niger* are known to produce cellulolytic, hemicellulolytic and ligninolytic enzymes. In their analysis of 94 representative fungal proteomes from Ascomycota, Basidiomycota, Chytridiomycota and Zygomycete fungi, Zhao et al. showed that more than half of the fungi analysed contained more than 300 CAZymes [3]. Regarding lignocellulose degradation, all of the analysed fungi have cellulases. In contrast, only 38% of a set of 1,500 bacterial genomes were reported to encode cellulases [4].

Yeasts are key organisms used in white biotechnology. The identification of some potential enzymatic strategies for biomass conversion was an important factor in the development of new and more efficient cell factories. In biorefinery approaches, biomass processing comprises three different steps: chemical and physicochemical pretreatment to make the biomass susceptible to the action of cellulolytic or hemicellulolytic enzymes; the enzymatic hydrolysis of the polysaccharide components of the pretreated biomass; and the fermentation of the resulting hexose and pentose sugars. Most traditional approaches separate the saccharification and the sugar fermentation steps [5]. Attempts have been made to merge these steps in second-generation bioethanol production using recombinant cellulolytic *Saccharomyces cerevisiae* strains [6], or by *S. cerevisiae* strains that have been engineered to degrade xylan and ferment xylose [7, 8]. However, they faced several obstacles on their way to industrialisation, due to the presence of inhibitory by-products in the biomass hydrolysate. Identifying new yeast species that are naturally capable of degrading plant biomass would make it possible to produce compounds of interest from lignocellulosic substrates.

In this way, Morais et al. isolated yeasts associated with rotting wood from four Atlantic Rain Forest sites in Brazil on a sugarcane bagasse hydrolysate based medium. They identified 44 strains belonging to 24 species that produced xylanase on a xylan medium with *Apiotrichum sporotrichoides*, *Aureobasidium pullulans*, *Saitozyma podzolica* and *Sugiyamaella xylanicola* showing the highest levels of enzyme activity [9]. In a search to identify new polysaccharide-degrading yeasts, CAZymes were predicted in 332 yeast species within the Saccharomycotina subphylum [10]. Twenty yeast species have more than 200 CAZymes and species from the Trichomonascaceae family, such as *Spencermartinsiella europaea* and *Blastobotrys proliferans*, contain the highest number with 204 and 203 predicted CAZymes, respectively. Of the eight Trichomonascaceae species that were studied further by Ravn et al., *Sp. europaea*, *Sugiyamaella lignohabitans* and *Blastobotrys peoriensis* possess endoxylanases of the glycosyl hydrolase family 10 (GH10), whereas *Blastobotrys mokoenaii* encodes a putative GH11 xylanase [10]. More recently, genes encoding xylanase from the GH11 family were also found in the *Blastobotrys illinoisensis* and *Blastobotrys malaysiensis* species [11]. By comparison, Zhao et al. found GH10 and GH11 enzymes in 52 and 41 out of the 94 fungal proteomes analysed, respectively [3].

In order to isolate new species of yeast capable of degrading hemicellulose, we explored novel niches, such as the gut of saproxylic insect larvae. In 2018, we isolated two strains, L1-24 and L2-36, from the feces of two cetonia larvae. As no identical sequences of the D1/D2 region of the ribosomal large subunit were found in public databases, we initially considered these isolates to represent a new species, which we named *Blastobotrys yvelinesensis* [12]. However, our phylogenomic analysis subsequently revealed that strain L1-24 shares 99.93% Average Nucleotide Identity (ANI) with strain CBS 14902, whose genome sequence was published in 2023 [13]. CBS 14902 has been described as the type strain of the species *Trichomonascus vanleenenianus* [14], clarifying the novel taxonomic assignation of our strains. *Trichomonascus* and *Blastobotrys* belong to the same genus: *Trichomonascus* is the sexual morph genus and *Blastobotrys* is the asexual morph genus. Despite the current “one fungus, one name” principle [15, 16], there is still controversy over which genus name should be retained. Visagie et al. [17] proposed the new combination *Blastobotrys vanleenenianus*, while Groenewald et al. [14] argued that *Trichomonascus* has priority over the asexual morph genus and therefore recommended retaining the genus name *T. vanleenenianus*. For this study, we used the name *T. vanleenenianus.*

Given that *Blastobotrys* spp. are among the top 25 yeasts in terms of the absolute CAZyme numbers [10], and the fact that L1-24 can grow on xylan [12], we investigated its potential to degrade hemicellulosic compounds using genomic, transcriptomic and physiological approaches. Our ultimate aim is to use this species as a yeast chassis or as a reservoir of valuable genes.

## Materials and methods

### Yeast strains and culture media

Experiments were performed using strains L1-24 (= CLIB 3782) and L2-36 (= CLIB 3783) of *T. vanleenenianus*. Both strains were isolated in 2018 from the feces of two beetle larvae collected from compost in Les Essarts-le-Roi (Yvelines, France). The strains have been deposited in the CIRM-Levures yeast collection (https://cirm-levures.bio-aware.com). Yeast strains were maintained on YPD medium (10 g/L yeast extract, 20 g/L Bacto peptone, 20 g/L glucose, 20 g/L agar).

### DNA and RNA isolation and sequencing

For DNA extraction, strain L1-24 was cultured for 32 h in 10 mL YPD medium at 28 °C, with shaking at 220 rpm. DNA extraction was based on an in-house protocol involving mechanical and chemical lysis, as previously described [18]. DNA samples were processed to generate libraries with the BGI Optimal DNA library Prep Kit (BGI, Hong Kong). After passing quality control, the libraries were sequenced with DNBseq technology using BGISEQ-500 instrument, generating paired-end reads of 2 × 150 bp. Low-quality reads were processed and filtered using fastp tool version 0.23.2 with default parameters [19].

For RNA extraction, strain L1-24 was cultured in 100 mL YNB-glucose (yeast nitrogen base 1.7 g/L, NH_4_Cl 5 g/L, glucose 10 g/L) and YNB-xylan (yeast nitrogen base 1.7 g/L, NH_4_Cl 5 g/L, xylan 10 g/L (Megazyme)) media at 28 °C with shaking at 220 rpm for 24 h. Cell cultures were divided in three samples and centrifuged 5 min at 4,500 rpm. The cell pellets were used to extract RNA with the RNeasy mini kit (Qiagen). RNAs were eluted in 40 µL of RNAse-free water. Quality control of the RNA preparations was performed through capillary electrophoresis with a 2100 Bioanalyzer system using an RNA 6000 Nano LabChip Kit (Agilent Technologies). Libraries were prepared from 100 ng total RNA using the NEBNext Ultra II Directional RNA Library Prep for Illumina (New England BioLabs) according to the manufacturer’s protocol. The libraries were sequenced using an Illumina NovaSeq 6000 instrument (Illumina, San Diego, CA, USA) in paired-end mode, generating 2 × 150 bp reads.

### Genome assembly and annotation

Genome assembly was generated using DNBseq reads with SPAdes assembler version 3.13.1 (parameter: -k 21,33,55,77,99,127) [20]. Ab initio gene detection was performed with Augustus version 3.5.0. The position of introns was selected from RNAseq data using bam2hints from Augustus software with parameter –intronsonly and filterIntronsFindStrand.pl. The resulting file was used as a hint for Augustus with parameter –species = yarrowia_lipolytica. tRNA genes were predicted with tRNAscan-SE v2.0.9 [21]. Manual curation with RNAseq data was performed as described previously [22]. The completeness of the annotation was evaluated with BUSCO v5.7.1 [23], using the saccharomycetes_odb10 lineage data set as the reference and the protein mode (2,137 proteins). Functional annotation was performed using the in-house software go-FAnnoT (https://github.com/hdevillers/go-fannot), which assigns annotations based on homologies found in the curated UniProt database (release 2024_02). The InterProScan version 5.71–102.0 tool was used to search for conserved domains within the 6,306 CDSs.

### Sequence data accession

The genome and transcriptome sequencing data have been deposited in the European Nucleotide Archive at EMBL-EBI under the accession number PRJEB90485, the assembly accession number is GCA_965637985.

### Protein prediction

Secretory proteins were predicted using the signalP-6.0 software (https://services.healthtech.dtu.dk/services/SignalP-6.0/). The subcellular localisation of these secretory proteins was then determined using the DeepLoc-2.0 software (https://services.healthtech.dtu.dk/services/DeepLoc-2.0/). Transmembrane domains and glycosylphosphatidylinositol (GPI) anchored proteins were predicted using the TMHMM 2.0 (https://services.healthtech.dtu.dk/services/TMHMM-2.0/) and the NetGPI-1.1 software (https://services.healthtech.dtu.dk/services/NetGPI-1.1/) respectively. We used dbCAN2 to identify CAZymes (https://bcb.unl.edu/dbCAN2/blast.php) [24].

### Transcriptome analysis

Low-quality RNA-seq reads (between 14.4 and 17.2 million reads per sample) were processed and filtered using the fastp tool version 0.23.2 with the default parameters [19]. The cleaned reads were then mapped onto the *T. vanleenenianus* L1-24 genome assembly using HISAT2 version 2.2.1 with the default parameters [25]. Filtering of secondary and supplementary mapped reads was performed using the samtools view tool [26]. Only segments that were properly aligned in paired-end were retained. The number of reads per protein-coding gene was counted using FeatureCounts version 2.0.3. Differential expression analysis was performed in the R environment (version 4.3.2) using the DESeq2 package version 1.42.1 [27]. A preliminary filtration of genes with low counts (no reads per kilobase in at least one out of the six libraries) was performed. Genes with low counts were manually investigated to identify high expression contrast between growth on glucose and xylan. The read counts of the remaining genes were then processed using the DESeq2 procedure. Replicates for both the glucose and xylan conditions were validated using factorial analysis with the ade4 package. Thresholds for differential expression were a log2 fold change of 2 and an adjusted p-value of 10^–3^.

### Phylogenomics

Thirty genomes, including the previously sequenced *T. vanleenenianus* CBS 14902 strain, were used for the phylogenomic analysis (Additional file 1). The first step was to identify a set of genes present in single-copy in each genome using BUSCO software version 6.0.0 [10.1093/nar/gkae987] with the parameters –mode genome –lineage_dataset saccharomycetes_odb10. Custom python script was used to parallelize the construction of multiple sequence alignments for each single-copy BUSCO protein family (*n* = 1076). Briefly, each family was aligned using MAFFT v7.526 [28] with default parameters and alignments were trimmed with ClipKIT v2.7.0 (–mode smart-gap) [29]. The resulting alignments were concatenated into a supermatrix containing 647,627 positions. A maximum-likelihood tree was inferred using IQ-TREE v3.0.1 [30] with parameters –ufboot 1000 (1000 ultra-rapid bootstraps replicates) –mset LG (restrict ModelFinder to test only LG protein evolution models). Finally, the tree was optimised using the “LG + F + I + R5” protein model. For each individual protein family, a ML tree was computed with IQ-TREE using the same parameters as above. Individual tree files were concatenated (one per line). Gene concordance factor (gCF) and site concordance factor (sCF) were obtained using IQ-TREE with parameters –gcf cat.trees (concatenated-trees file) and –scf 1000 (1000 quartets). FigTree v1.4.4 (http://tree.bio.ed.ac.uk/software/figtree) was used to visualize the tree.

### Genome Average Nucleotide Identity (ANI)

ANI was calculated using OrthoANIu algorithm [31].

### Transformation of L1-24 with a DsRed reporter cassette

A reporter tool consisting of the DsRed coding sequence cloned downstream from the *B. raffinosifermentans XYL1* promoter was constructed. For this purpose, the DsRed sequence was amplified using primers DsRed_For (CCGGATCCCACAATGAGTGCTTC) and DsRed_Rev (CCGCGGCCGCTTACAAGAACAAGTGGTGTC) and cloned at the *Bam*HI and *Not*I sites of the previously reported pBS-SA-p*XYL1*-eYFP-PHO5t [12] to generate the pBS-SA-p*XYL1*-DsRed-PHO5t. In parallel, the *hph* resistance marker contained in the pARE12-p*XYL1*-eYFP plasmid was replaced by the *nat* marker using *Eco*RI restriction, resulting in the pARE14-p*XYL1*-eYFP plasmid. Finally, the p*XYL1*-DsRed-PHO5t cassette was subcloned in the latter plasmid as a *Age*I/*Apa*I fragment to obtain the pARE14-p*XYL1*-DsRed-PHO5t plasmid (Additional file 2). An *Asc*I restriction fragment was used for transformation of the L1-24 strain using the same protocol as for *B. raffinosifermentans* [12] and selection was performed on YEA + 100 µg/mL of nourseotricin.

## Results

### Genome sequencing and phylogenetic placement

The nuclear genome of L1-24 (CLIB 3782) was assembled into 116 scaffolds of size larger than 5 kb, with a total size of 14.9 Mb (Table 1; project accession number PRJEB90485, assembly accession number GCA_965637985). By comparison, the size of the *T. vanleenenianus* CBS 14902 genome, which had previously been sequenced, was 15.3 Mb (Additional file 1). The G + C content is 49.4%, the N50 is 254,743 kb (L50 = 19). Six telomeric repeats TAGAACGGG were found at the end of scaffolds TRVA0_038S, TRVA0_071S, TRVA0_091S, and TRVA0_0113S, and at the beginning of scaffolds TRVA0_076S and TRVA0_108S (Additional file 3). Two additional telomeric repeats were found in scaffolds smaller than 5 kb, suggesting that L1-24, like other *Blastobotrys* species, may have 4 chromosomes [32].

**Table 1:** Genome features of *T. vanleenenianus* L1-24 and comparison with the genome of the three most closely related *Blastobotrys* species

Species	*T. vanleenenianus*	*T. vanleenenianus*	*B. illinoisensis*	*B. malaysiensis*	*B. mokoenaii*
Strain	L1-24	CBS 14902	NRRL YB-1343	NRRL Y-6417	NRRL Y-27120
Total sequence length	14.9 Mb	15.3 Mb	14.3 Mb	15.8 Mb	13.7 Mb
Number of scaffolds	117	635	7	2.553	-
N50 scaffolds	254.7 kb	71.4 kb	3,252 kb	118.5 kb	-
L50 scaffolds	19	67	2	40	-
Number of contigs	125	635	7	3.421	333
N50 contigs	235.5 kb	49.8 kb	3,252 kb	29.4 kb	249.2 kb
L50 contigs	20	89	2	159	15
GC percent	49.4	49.5	51	50.5	49
Genome coverage	82.2x	181.4x	350.0x	34.8x	73.0x
BUSCO complete	2025	2017	2022	1987	2026
BUSCO complete—single copy	2014	2006	2011	1958	2012
BUSCO complete—duplicated	11	11	11	29	14
BUSCO fragmented	51	54	52	73	45
BUSCO missing	61	66	63	77	66

BUSCO lineage: saccharomycetes_odb10

BUSCO number of proteins: 2137

**Fig. 1:**
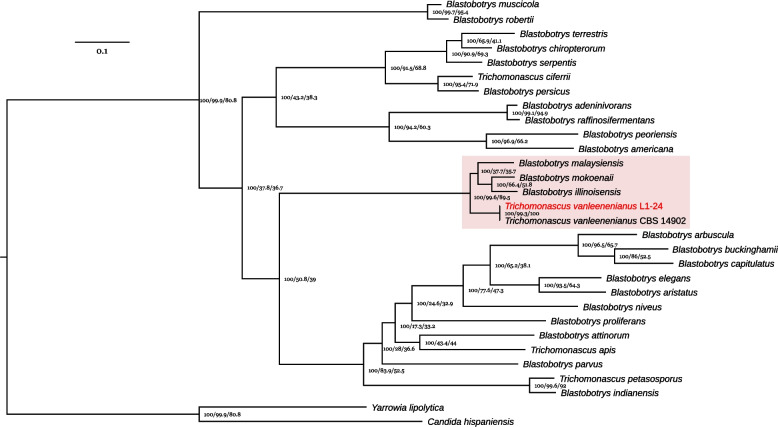
Phylogenetic relationships between *Blastobotrys* and *Trichomonascus* species. The maximum-likelihood phylogenetic tree, which is rooted using *Yarrowia lipolytica* CLIB 122 and *Candida hispaniensis* CBS 9996, is based on a concatenated alignment of 1,076 conserved single-copy BUSCO proteins. The support values at the nodes represent the ultrafast bootstrap (UFB), gene concordance factor (gCF), and site concordance factor (sCF), respectively (UFB/gCF/sCF). *T. vanleenenianus* L1-24 is highlighted in red, and the four closely related species have a pink shaded background

Of the 34 *Blastobotrys* and five *Trichomonascus* species that have been described, genome sequence was available for 28 of them by the end of 2025 (Additional file 1). However, only the annotation of *T. ciferrii* CBS 4856 genome is available in genomic databases. A ML-phylogenenetic tree (Fig. 1) based on 1076 conserved single-copy BUSCO protein families was reconstructed and confirmed that L1-24 belongs to the species *Trichomonascus vanleenenianus,* with support values 100/99.3/100 for ultrafast bootstrap (UFB), gene concordance factor (gCF), and site concordance factor (sCF), respectively (UFB/gCF/sCF). *T. vanleenenianus* groups with three closely related species, including *B. illinoisensis, Blastobotrys mokoenaii,* and *Blastobotrys malaysiensis*. ANI analysis revealed 99.93% nucleotide identity between L1-24 and CBS 14902, the type strain of *T. vanleenenianus*. The nucleotide identity with the three most closely related *Blastobotrys* species varied from 78.3 to 79.0% (Additional file 4).

### Genome annotation of strain L1-24

Genome annotation led to the prediction of 6,306 CDSs and 75 pseudogenes, mainly corresponding to remnants of transposable elements (LINE, LTR-retrotransposon of the Ty3-gypsy family, Tc1-mariner). This number is slightly higher than the 6,116 CDS predicted for *B. raffinosifermentans* [33] but lower than the 6,913 proteins encoded by the *T. ciferrii* genome [34] (Table 2). By way of comparison, 6,177, 7,209 and 6,069 protein-coding genes were predicted in *B. illinoisensis*, *B. malaysiensis* and *B. mokoenaii*, respectively [11]. Following the functional annotation of L1-24 protein-coding genes, it was found that 333 of the 6,306 CDS (~ 5%) had either no homologous proteins in *S. cerevisiae* or no conserved domains.

**Table 2:** Annotated features of *T. vanleenenianus* compared to other Saccharomycotina species

Species	*S. cerevisiae*	*Y. lipolytica*	*G. candidum*	*B. raffinosifermentans*	*T. ciferrii*	*T. vanleenenianus*
Strain	S288c	E150	CLIB 918	LS3	CBS 4856	L1-24
Chromosome number	16	6	8	4	4	4
Genome						
Ploidy	n	n	-	n	n	n
Size (Mb)	12.1	20.5	24.8	11.8	20.5	14.9
Average G + C content (%)	38.3	49.0	48.0	48.1	47.5	49.4
CDS						
Total CDS	5.769	6.449	6.804	6.116	6.913	6.306
i-genes	287	984	2.415	703	-	1.706
Introns	296	1.119	3.830	914	-	2.362
tRNA	274	510	352	147	-	110
rDNA clusters	1 (internal)	6 (subtelomeric)	-	1 (internal)	-	1 (internal)
Dispersed 5S rRNA genes	0	116	-	30	-	36

Data on *S. cerevisiae*, *Blastobotrys raffinosifermentans* and *Y. lipolytica* were obtained from Kunze et al. [33], except for the introns data, which were obtained from Neuvéglise et al. [36]

Data on *G. candidum* were obtained from Morel et al. [35] and data on *T. ciferrii* from Mixao et al. [34]

A huge number of introns was observed, i.e., 2,362 introns in CDS of 1,706 genes, 176 in 5′UTR or 3′UTR and 44 in 53 miscellaneous RNA. Nearly 25% of the genes contain at least one intron, which places *T. vanleenenianus* between *Y. lipolytica* and *Geotrichum candidum*, the two species with the highest percentages of intron-containing genes in hemiascomycetous yeasts, with 15% and 35% respectively [35, 36]. In *B. raffinosifermentans*, a total of 914 introns were identified in 703 genes [33]. The patterns of the 5' and 3' intron boundaries, and of the branching point, as well as their length distribution, were estimated for the 2,539 introns (Additional file 5).

A single ribosomal DNA cluster has been found at the 3’ end of contig TRVA0_081. The 18S rRNA gene (1,763 bp) and the 5.8S rRNA gene (158 bp) are complete, whereas the 26S rRNA gene (2,559 bp) is partial. As in *B. raffinosifermentans* strain LS3 [33], the 5S rRNA gene is dispersed throughout the genome. Twenty-one complete copies have been annotated in the 116 scaffolds, and a further 15 copies were found in contigs smaller than 5 kb. Interestingly, all of the 5S rRNA genes were found in tandem with tRNA genes, separated by 1–3 bp. There are 110 nuclear tRNA genes, 16 of which are arranged in tandem. Twenty-two tRNA genes are fused with 5S genes. Fusions between tRNA and 5S rRNA genes have previously been reported for *Y. lipolytica*. The authors demonstrated that TFIIIA, an essential DNA binding protein required for transcription of 5S rRNA by RNA polymerase III, could be deleted since half of the 5S copies were transcribed in fusion with tRNA [37]. In L1-24, all the 5S rRNA genes are arranged in tandem with the tRNA genes, which suggests that *TFIIIA* may be either absent or defective. A BLAST search showed that the gene exists (TRVA0_062S00430), which has a 5’ extension of approximately 600 bp compared to closely related homologues. Furthermore, RNA-seq data revealed that TRVA0_062S00430 is expressed under both glucose- and xylan-growth conditions.

The MAT locus was identified through synteny with *B. raffinosifermentans* LS3, using the *SLA2* gene, which is located downstream of *MATalpha1* [33]. The gene TRVA0_001S07525 in L1-24, upstream of *SLA2* (TRVA0_001S07547)*,* is a *MATalpha1* transcriptional activator and there is no *MATalpha2* gene. The *MATalpha1* amino acid sequence contains a pfam04769 conserved domain that corresponds to a MATalpha_HMGbox domain. The *MAT* locus region is conserved in synteny in the three most closely related species of *T. vanleenenianus* species (Additional file 6)*.* Surprisingly, only *MATalpha1* was found in these yeasts. So far, strains of the opposite mating type have not yet been identified. A chromosomal rearrangement occurred in the ancestor of these four species as evidenced by the broken synteny with *APN2*, whose position is however conserved from *B. raffinosifermentans* LS3 and *T. ciferrii* to *Y. lipolytica* [33].

For efficient sexual reproduction, proteins involved in mating, such as pheromone receptors and pheromone maturation endoproteases, should be present in the genome. Indeed, they were identified based on their annotation or through BLASTp analysis using *B. raffinosifermentans* LS3 proteins (Additional file 7). A single putative mating pheromone alpha (MF-alpha) was identified following a search for repeated Kex2 recognition sites (KR/RR) in secretory proteins that had no known function. The retained candidate, TRVA0_001S07646, contains twelve repetitions of the SN/KGGDLPIYGEPGW peptide. These repetitions are delimited by KR dipeptides and could undergo further processing by the Ste13 endoprotease after the proline residue [38]. The resulting IYGEPGW peptide displays five amino acid residues that are conserved in the *Y. lipolytica* MF-alpha GYGEPNW sequence [33]. However, no mating pheromone a (MFA1/2) has been detected in the genome of L1-24, raising questions about the sexual reproduction of this species.

Structural annotation of the genome allowed us to predict the function and localization of proteins. To determine xylan-degrading capabilities, it is essential to identify secreted proteins, particularly CAZymes.

### Prediction of secretory proteins

**Fig. 2:**
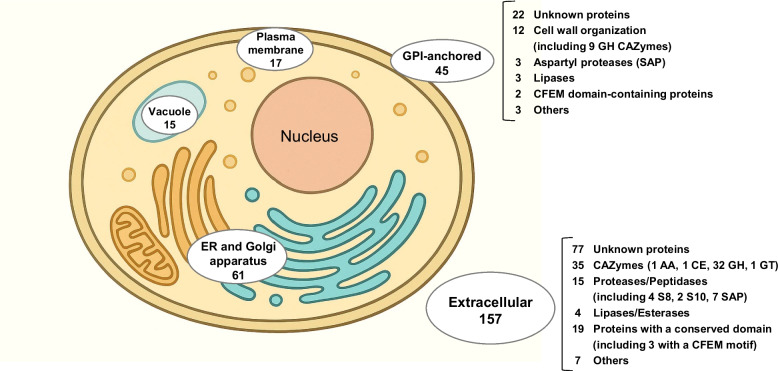
Subcellular localisation of the 295 predicted secretory proteins. Predictions were performed using softwares available at the Technical University of Denmark (https://services.healthtech.dtu.dk/services/). The yeast cell was generated using ChatGPT (OpenAI, 2025). The functional classes of GPI-anchored and extracellular proteins are detailed on the right

The signalP6.0 software was used to identify proteins with a N-terminal signal sequence. Of the 6,306 predicted proteins, 295 are potentially secretory proteins. The subcellular localisation of these secretory proteins was then determined using the DeepLoc-2.0 software: 61 proteins were predicted to be addressed to the endoplasmic reticulum or the Golgi apparatus, 17 in the plasma membrane and 15 in the vacuole. Of the remaining 202 proteins, 45 were predicted to be exposed on the cell surface by the addition of a GPI anchor. The remaining 157 proteins are extracellular (Fig. 2; Additional file 8).

Of the 202 secreted or surface-anchored proteins, 99 correspond to proteins whose function is unknown and which lack a conserved domain. The second largest group comprises 9 cell wall-associated proteins and 35 extracellular proteins (Fig. 2), which are enzymes active on polysaccharides, i.e., CAZymes (Additional file 9). Some of these are similar to proteins in *S. cerevisiae* that act on β−1,3-glucan or chitin polymers and contribute to the expansion and remodelling of the yeast cell wall. These enzymes consist of exo-β−1,3-glucosidases and endo-β−1,3-glucosidases that belong to the GH5_9/GH132 or GH17/GH81 CAZyme families, respectively. Three extracellular endochitinases of the GH18 family are also predicted. Six enzymes responsible for linkages between β−1,3-glucans (GH72) or between β−1,3-glucans and chitin (GH16) were also identified.

The third major class of the 202 secreted or surface-anchored proteins comprises proteases belonging to the eukaryotic aspartyl protease family (SAP), which contains ten members, three of which are localized in the cell wall. All of these proteins contain the conserved domain pfam00026, which is characteristic of this class. The two aspartic acid (D) residues, which play a key catalytic role in the active site, are conserved (Additional file 10). Eight other peptidases, corresponding to serine proteases of the S8 subtilisin-like subfamily or to serine carboxypeptidase of the S10 subfamily are predicted as extracellular proteins (Fig. 2; Additional file 8) [39, 40].

Five lipases were identified in L1-24, and contained a pfam03583 conserved domain [41]. Three of them are likely to be GPI-anchored (Fig. 2; Additional file 8). In comparison, only one homologous lipase gene was found in *B. raffinosifermentans* and six lipases were found to be encoded by the *T. ciferrii* genome: two are putatively retained on the cell surface by a GPI-anchor and four are secreted. The alignment of these twelve proteins showed the presence of conserved motifs and specific protein extensions in the N- or C-terminal regions. The growth of *T. vanleenenianus* on the tributyrin medium was accompanied by the formation of a thin halo around the colonies, which indicates lipase activity (Additional file 11).

### Identification of CAZymes and comparison with those of other *Blastobotrys* species

A total of 192 CAZymes have been identified, divided between the Auxiliary Activities (AA: *n* = 18), Carbohydrate-Binding Modules (CBM: *n* = 2), Carbohydrate Esterases (CE: *n* = 3), Glycoside Hydrolases (GH: *n* = 104), Glycosyl Transferases (GT: *n* = 63) and Polysaccharide Lyases (PL: *n* = 2) families [42]. By way of comparison, 144, 180 and 214 CAZymes were predicted on the MycoCosm platform in *S. cerevisiae* S288C, *B. raffinosifermentans* LS3 and *Trichomonascus petasosporus* NRRL YB-209, respectively [43]; while 204, 221 and 213 CAZymes are detected in *B. illinoisensis*, *B. malaysiensis* and *B. mokoenaii*, respectively [11]. Other species, such as *Lipomyces* spp., generally possess more CAZymes, with up to 279 in *Lipomyces tetrasporus* NRRL Y-27496.

Plant cell walls are mainly composed of celluloses and hemicelluloses. We have therefore focused on the main families of GH enzymes involved in the degradation of these polymers including cellulases and hemicellulases (see below).

Cellulosic enzymes such as β−1,4-endoglucanases, exoglucanases/cellobiohydrolases and β-glucosidase, which are required for cellulose degradation, are grouped into eight GH families (GH1, GH3, GH5, GH6, GH7, GH12, GH13 and GH45) [44]. Of the seven GH5 family proteins in *T. vanleenenianus*, three retained our attention. TRVA0_002S01442, which is secreted, is predicted to belong to the GH5_5 subfamily (Additional file 9). The GH5_5 subfamily comprises enzymes with endo-β−1,4-glucanase activities, with cellulose serving as the substrate [45]. Proteins of this family are rare in Saccharomycotina and have only been predicted in *L. tetrasporus* in the MycoCosm database, which includes 216 genomes of 153 species in the Saccharomycotina subphylum. *B. mokoenaii*, *B. malaysiensis* and *B. illinoisensis* contain one, two and four homologous proteins, respectively, divided into two groups (Additional file 12).

**Fig. 3:**
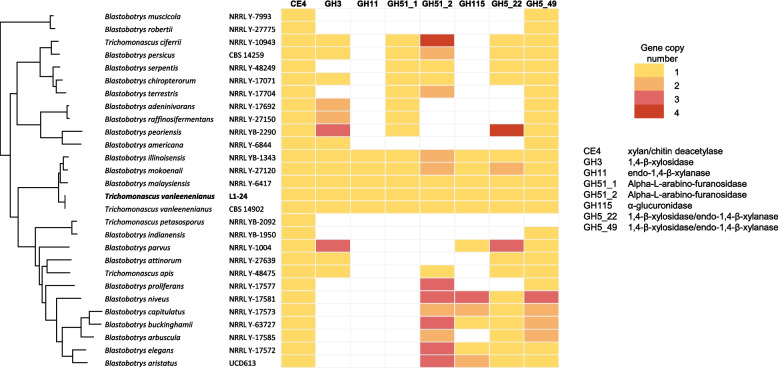
Occurrence of genes coding for xylanolytic CAZyme families in *T. vanleenenianus* L1-24 (in bold) and all *Blastobotrys* and *Trichomonascus* species included in the phylogenomic tree presented in Fig. 1. The CAZyme families are listed at the top, and the number of genes in each family is colour-coded. Functions associated with the CAZyme families are shown on the right

Two other *T. vanleenenianus* enzymes belong to the GH5_22 (TRVA0_001S00320) and GH5_49 (TRVA0_001S10418) subfamilies, respectively (Additional file 9). The GH5_22 subfamily comprises enzymes that exhibit cellulose endo-β−1,4-glucosidase, xylan endo-β−1,4-xylosidase or xylan exo-β−1,4-xylosidase activities and may therefore have cellulose or xylan as substrates [45]. The *T. vanleenenianus* TRVA0_001S10418 protein is predicted to be located in the cytoplasm as the GH5_49 member does. Although no activity has been documented for this subfamily in the CAZyme database, Ravn et al. recently reported endo-xylanase activity for the heterologously expressed GH5_49 protein from the xylanolytic yeast *Wickerhamomyces canadensis* [46]. The GH5_22 and GH5_49 enzymes are prevalent in the *Blastobotrys/Trichomonascus* genus (Fig. 3) and, more broadly, in the phylum Saccharomycotina.

The composition of hemicelluloses depends on the sugars that form their backbones. For example, xylan contains β−1,4-linked D-xylose, glucomannan contains β−1,4-linked D-mannose and D-glucose, and xyloglucan contains β−1,4-linked D-glucose. With regard to the GH families of xylanolytic enzymes, *T. vanleenenianus* harbours a member of most of them (Fig. 3 and Additional file 9).

The main activity required for xylan degradation, β−1,4-endoxylanase (Xyn1), is mainly found either in the GH10 or the GH11 family. Enzymes from these families are widespread across species in the Pezizomycotina subphylum, whereas they are rare in Saccharomycotina. One *T. vanleenenianus* predicted secretory protein, TRVA0_032S00122, belongs to the GH11 family. Homologues are exclusively present in the three closest species among the 28 *Blastobotrys/Trichomonascus* species whose genome is available (Fig. 3). These four proteins are highly conserved with each other and with xylanases from *Aspergillus spp.* (Additional file 13). Their capacity to hydrolyse xylan has previously been reported [10–12]. In Saccharomycotina, xylanases of the GH10 family have been reported in several *Scheffersomyces* species (9 species in MycoCosm) and in *Sugiyamaella americana*, but no xylanase of the GH11 family had been described prior to Ravn's studies [11, 43, 46].

The GH3 family comprises enzymes with exo-1,4-β-xylosidase activity (Xyl1). Six GH3 enzymes have been identified in the *T. vanleenenianus* genome. Two of these are predicted to be secreted, but only one, TRVA0_006S02916, contains the conserved PLN03080 domain found in β-xylosidases [41]. Similar proteins are also present in the three closely related species of *T. vanleenenianus,* as well as in *B. adeninivorans* and *B. raffinosifermentans,* which have two copies of β-xylosidases (Fig. 3) [12]. This suggests that these species can grow on xylobiose.

An endoxylanase activity was recently characterized in a GH30_7 subfamily protein from *Talaromyces leycettanus,* a member of the Pezizomycotina [47]. The species *B. illinoisensis*, *B. mokoenaii* and *B. malaysiensis*, encode enzymes belonging to the GH30 family [11, 48]. In *T. vanleenenianus*, TRVA0_091S00144, the only GH30 family member, belongs to subfamily GH30_5, which comprises exo-β−1,6-galactobiosidases (Additional file 9). Notably, this enzymatic activity is absent from Saccharomycotina according to the MycoCosm database and may play a role in the degradation of arabinogalactan polymers, such as those found in larch wood.

Depending on the plant species, monomers such as D-galactose, D-xylose, L-arabinose and D-glucuronic acid can be branched to the backbone. Therefore, several accessory enzymatic activities are therefore required to degrade these branched polysaccharides. L-arabinose is the most abundant branching residue in cereal xylans [49]. Alpha-L-arabinofuranosidases are found in the GH43, GH51, GH54 or GH62 CAZyme families. Two *T. vanleenenianus* secretory proteins belong to the subfamilies GH43_6 and GH43_24, TRVA0_020S02520 and TRVA0_042S00870, respectively (Additional file 9). The first one has an arabinan endo-1,5-α-L-arabinanase conserved domain cd18831, suggesting potential activity against arabinan, a plant polysaccharide that is abundant in sugar beet, various seeds and roots. The second one presents a galactan 1,3-β-galactosidase conserved domain (cd18821 in CDD). Neither of these enzymes is capable of degrading hemicellulose.

**Fig. 4:**
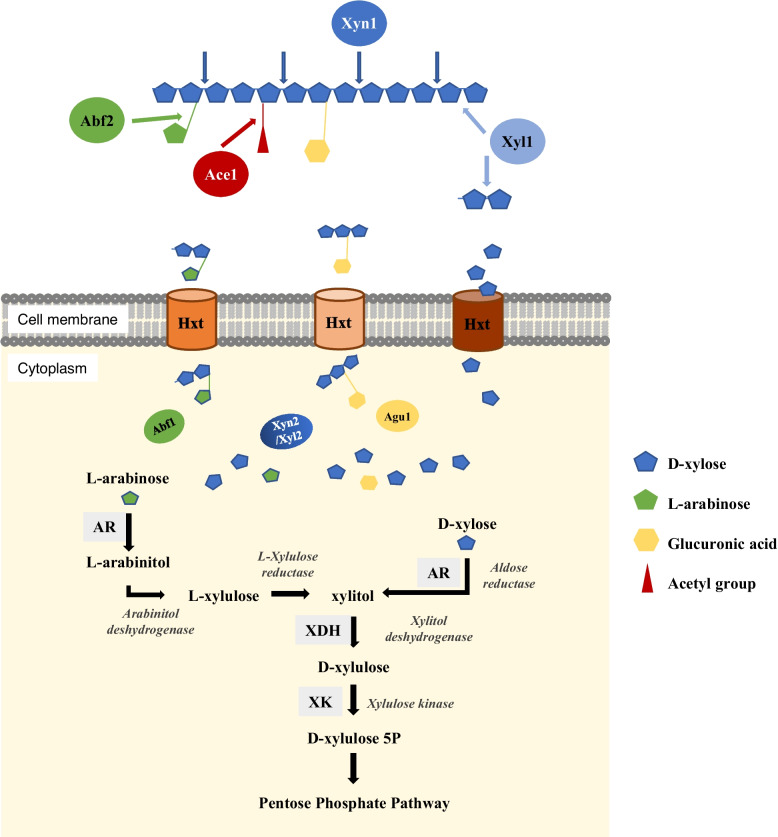
Xylan degradation and assimilation of the resulting pentoses by *T. vanleenenianus*. The extracellular and cytoplasmic enzymatic activities that lead to xylan saccharification are shown in coloured discs. Transporters of simple sugars and oligoxylosides are represented by cylinders within the cell membrane. The simple sugars are depicted by coloured symbols depicted on the right. The enzymes involved in the subsequent modification of L-arabinose (left) and D-xylose (right) until the D-xylulose 5P are shown in grey squares. AR: aldose reductase; XDH: xylitol dehydrogenase; XK: xylulose kinase

Two GH51 family proteins are also present in *T. vanleenenianus*, in the subfamilies GH51_1 (TRVA0_020S02498) and GH51_2 (TRVA0_067S00166). The latter is secreted (Figs. 3 and 4). These sequences are similar to α-L-arabinofuranosidases and may therefore be involved in arabinoxylan degradation. At least one member of the GH51_1 and GH51_2 subfamilies is present in the three closely related species, and a second copy of the GH51_2 α-L-arabinofuranosidase is present in *B. illinoisensis* and *B. mokoenaii* (Fig. 3). These enzymes are present in several *Blastobotrys* species, with *B. adeninivorans* and *B. raffinosifermentans* containing one GH51_1 α-arabinofuranosidase, and *T. ciferrii* containing four GH51_2 enzymes.

No members of the GH54 or the GH62 families were predicted in the *T. vanleenenianus* genome in contrast to *B. mokoenaii*, which has an arabinofuranosidase of the GH62 family [48].

The second debranching activity is the cleavage of α-glucuronic acid, and the enzymes that enable this hydrolysis belong either to the GH67 family or to the GH115 family. One α-glucuronidase, TRVA0_064S00386, belonging to the GH115 family is encoded by *T. vanleenenianus*. A second sequence, TRVA0_088S00144, is annotated as a pseudogene due to a mismatch in the coding sequence*.* Interestingly, no peptide signal was detected for the GH115 α-glucuronidase, suggesting that the xylooligosaccharides to which α-glucuronic acids are attached can enter the cells for degradation (Fig. 4). *B. illinoisensis*, *B. malaysiensis* and *B. mokoenaii* also contain an intracellular member of the GH115 family (Fig. 3 and Additional file 9). In contrast to these species, no members of the GH67 family were identified in *T. vanleenenianus* L1-24 or CBS 14902 [11].

A gene encoding an enzyme from the Carbohydrate Esterase family 4 (CE4), which includes acetylxylan esterases and chitin deactylases, is predicted in the *T. vanleenenianus* genome (TRVA0_039S00210, Additional file 9). The deacylation of either xylan or chitin could favour the subsequent degradation of the backbone of these two polysaccharides. All species in the genus *Blastobotrys* contain a homologue of TRVA0_039S00210 (Fig. 3). No gene that encodes a feruloyl esterase was detected in the *T. vanleenenianus* genome (CE1).

### Differential expression in glucose and xylan growth conditions

**Fig. 5:**
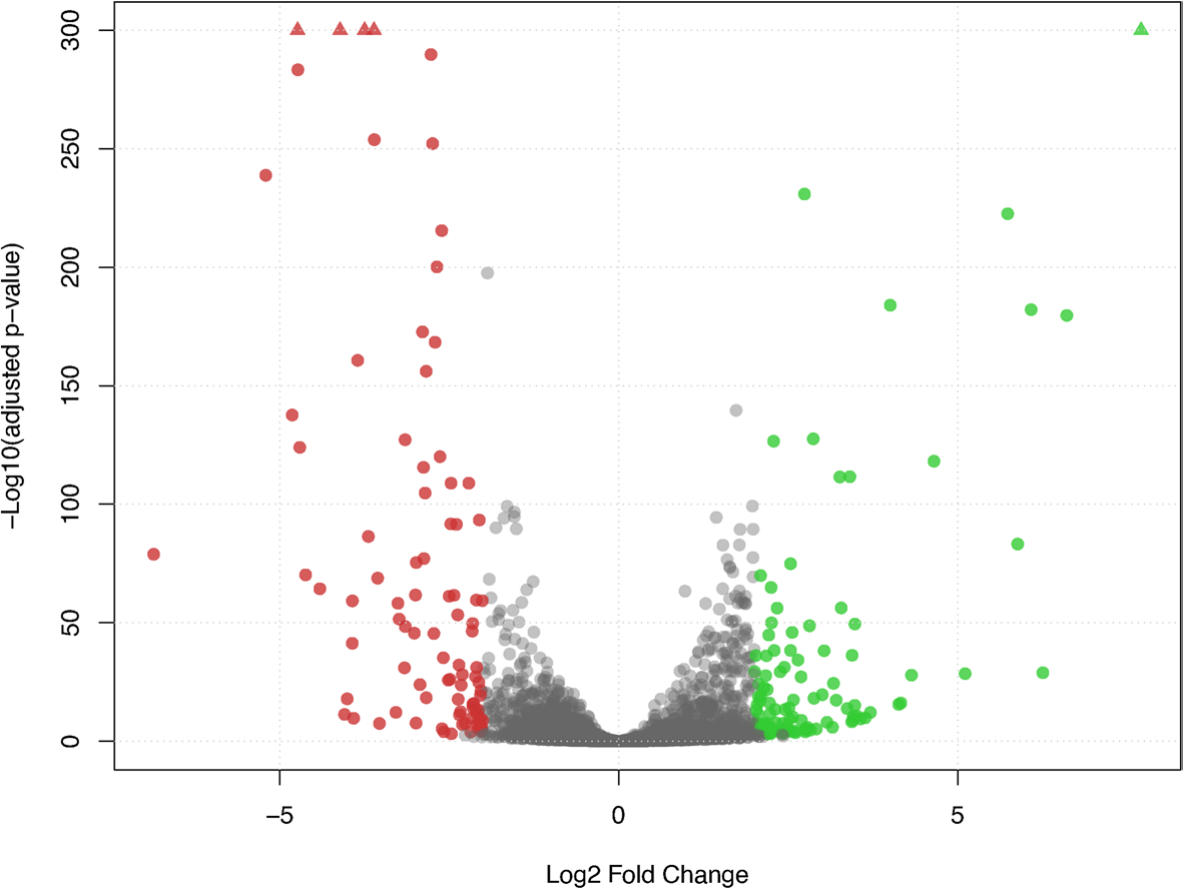
Volcano plot of differential expression (DE) in glucose and xylan growth conditions. The log2 Fold Change indicates the mean expression level for each gene. Each dot represents one gene. Black dots represent genes with no significant DE. Green and red dots represent genes that are up- and down-regulated, respectively. The threshold for log2FC was 2, and for the adjusted p-value it was 10^–3^

To confirm the involvement of some of the predicted CAZymes in hemicellulose degradation, a RNAseq experiment was performed and genes expressed in xylan growth conditions were compared with their expression in glucose. First, we applied a cutoff of one read per kilobase to the CDSs in order to filter out genes with low expression. After filtering, 791 protein-coding genes were removed and the expression of the remaining 5,572 genes was compared. Then, we used as cutoffs a log2 fold change (log2FC) of 2 and an adjusted p-value of 0.001. A total of 194 genes showed a significant change in expression with 102 being up-regulated and 92 being down-regulated under xylan growth conditions relative to the glucose growth conditions used as the control (Fig. 5 and Additional file 14).

The sixteen genes most strongly induced by xylan showed induction factors (i.e., the ratio between the number of reads in xylan versus glucose) ranging from 11 to 200 (Table 3). The most highly induced gene (TRVA0_021S00496), which is also one of the most highly expressed, encodes an aldose reductase that catalyses the NAD(P)H-dependent reduction of xylose to xylitol and arabinose to arabinitol, the first reactions in the xylose or arabinose utilization pathways (Fig. 4). Two other enzymes are required to assimilate xylitol: a xylitol dehydrogenase (XDH) and a xylulo kinase (XK). *T. vanleenenianus* has three *XDH* genes (TRVA0_002S05446, TRVA0_006S02608 and TRVA0_002S02608) and one *XK* gene (TRVA0_057S00782). TRVA0_002S05446 and TRVA0_006S02608 were induced in xylan as well as the *XK* gene, their induction factor was 10.2, 7.8 and 9.2, respectively (ranks 23, 31 and 26 in the log2FC column; Additional file 14).

**Table 3:** Genes overexpressed in xylan medium compared to glucose medium

Gene Id	Mean of reads in Glucose	Mean of reads in Xylan	Induction factor	Log2 fold change	*p*-adjusted values	Functional annotation
TRVA0_021S00496	22.0	4409.7	200.4	7.71	0	similar to uniprot|P38715 Saccharomyces cerevisiae YHR104W GRE3 aldose reductase
TRVA0_001S00320	15.3	1434.7	93.6	6.61	2.12E-180	1,4-β-xylosidase/endo-1,4-β-xylanase (GH5_22)
TRVA0_015S00562	2.7	196.0	73.5	6.26	1.23E-29	similar to uniprot|P39522 Saccharomyces cerevisiae YJR016C ILV3 dihydroxyacid dehydratase
TRVA0_022S00650	19.3	1256.7	65.0	6.08	8.46E-183	similar to uniprot|P54862 Saccharomyces cerevisiae YOL156W HXT11 probable glucose transporter
TRVA0_064S00386	9.3	530.7	56.9	5.89	6.40E-84	α-glucuronidase (GH115)
TRVA0_007S02322	31.3	1594.0	50.9	5.74	2.86E-223	similar to uniprot|P54854 Saccharomyces cerevisiae YDL245C HXT15 probable glucose transporter
TRVA0_013S02234	4.7	155.3	33.3	5.11	2.96E-29	dihydrodipicolinate synthase family protein
TRVA0_006S02916	27.3	658.3	24.1	4.65	7.19E-119	1,4-β-xylosidase (GH3)
TRVA0_041S01046	7.0	134.3	19.2	4.32	1.32E-28	similar to uniprot|P14065 Saccharomyces cerevisiae YOR120W GCY1 glycerol dehydrogenase involved in glycerol catabolism under microaerobic conditions
TRVA0_020S02542	4.7	80.0	17.1	4.16	7.24E-17	similar to uniprot|P13181 Saccharomyces cerevisiae YLR081W GAL2 GAL2 is a facilitated diffusion transporter required for both the high-affinity galactokinase-dependent and low-affinity galactokinase-independent galactose transport processes
TRVA0_009S00254	4.7	77.3	16.6	4.13	2.82E-16	highly similar to uniprot|Q02196 Saccharomyces cerevisiae YKL001C MET14 catalyzes the synthesis of activated sulfate
TRVA0_060S00144	79.3	1218.0	15.4	4.01	1.10E-184	similar to uniprot|P39676 Saccharomyces cerevisiae YGR234W YHB1 is involved in NO detoxification in an aerobic process, termed nitric oxide dioxygenase (NOD)
TRVA0_025S00870	52.3	643.0	12.3	3.71	7.42E-13	similar to uniprot|P32907 Saccharomyces cerevisiae YNR002C ATO2 transporter protein required for ammonia export
TRVA0_027S00364	4.0	47.7	11.9	3.64	1.36E-10	similar to uniprot|P30605 Saccharomyces cerevisiae YDR497C ITR1 major transporter for myo-inositol
TRVA0_041S01068	4.0	45.3	11.3	3.57	3.95E-10	YjbQ family protein may have thiamin phosphate synthase activity
TRVA0_010S01904	5.0	55.0	11.0	3.51	2.24E-11	similar to uniprot|P00175 Saccharomyces cerevisiae YML054C CYB2 catalyzes the oxidation of (S)-lactate (L-lactate) to pyruvate with subsequent transfer of electrons to cytochrome c
TRVA0_032S00122	0.0	1540.7	nd	nd		endo 1,4-β-xylanase (GH11)
TRVA0_088S00122	2.7	504.7	189.3	7.6		similar to uniprot|P53048 Saccharomyces cerevisiae YGR289C MAL11 high-affinity uptake of alpha-glucosides such as maltose, turanose, isomaltose, alpha-methylglucoside, maltotriose, palatinose, trehalose, melezitose and glucose
TRVA0_015S02278	1.3	140.0	105	6.7		similar to uniprot|P23585 Saccharomyces cerevisiae YMR011W HXT2 high-affinity glucose transporter
TRVA0_088S00188	0.0	114.7	nd	nd		similar to uniprot|A0A161HGA1 Sugiyamaella lignohabitans AWJ20_4583 glycoside hydrolase family 35 protein

Three genes highly expressed in xylan encode proteins of the CAZyme families GH3, GH5_22 and GH115 with log2FC ranks of 8, 2 and 5, respectively (Table 3). A fourth up-regulated gene (TRVA0_019S01376, log2FC rank of 19) corresponds to the predicted α-arabinofuranosidase from the GH51_1 family (Fig. 3). The up-regulation of these four genes further supports their predicted role in xylan degradation, particularly for the GH5_22 protein, which can act as an intracellular xylan exo- or endo-β−1,4-xylosidase. The expression of two genes encoding proteins similar to *S. cerevisiae* Hxt11 and Hxt15 transporters strongly increased in xylan (ranks 4 and 6; Table 3). These transporters could facilitate the entry of xylose, as well as arabinose- and glucuronic acid-substituted xylooligosaccharides into cells, which will be hydrolysed by the GH115 (Agu1) and GH51_1 (Abf1) enzymes predicted to be intracellular (Fig. 4). The GH51_1 enzyme releases L-arabinose, which is subsequently catabolized in xylitol through the activity of three enzymes: aldose reductase (TRVA0_021S00496), L-arabinitol 4-dehydrogenase (TRVA0_006S02608) and L-xylulose reductase (TRVA0_002S05446). The three genes are overexpressed in xylan (log2FC ranks of 1, 31 and 23, respectively; Additional file 14).

Four genes, which were discarded due to their weak expression in glucose, completed the list of highly expressed genes in xylan (Table 3). *XYN1*, which encodes the GH11 endoxylanase, was the most highly expressed gene; no transcript of this gene was detected in glucose. We previously detected Xyn1 in the culture medium of L1-24 under similar growth conditions [12]. Two of the four discarded genes encode transporters. Interestingly, the fourth gene encodes a CAZyme of the GH35 family, for which no xylan-degrading activity has yet been observed.

### Monitoring xylan degradation and sugar uptake via p*XYL1*-Driven DsRed expression

**Fig. 6:**
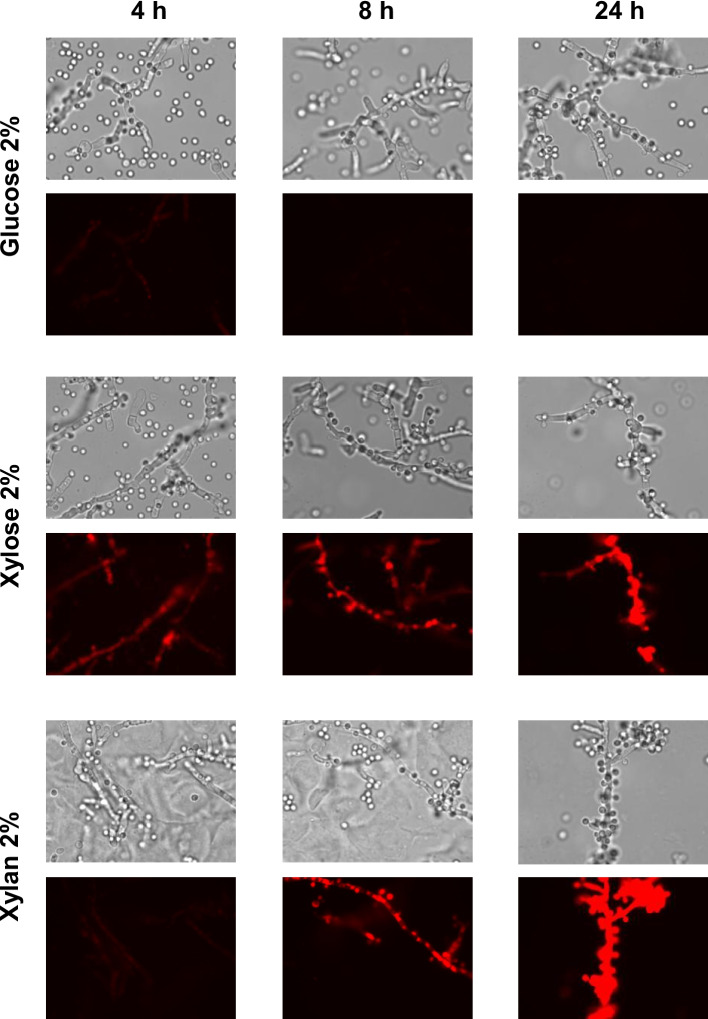
Monitoring of the time-course induction of the *XYL1* promoter using RedStar fluorescence. *T. vanleenenianus* cells were grown in media containing 2% glucose, 2% xylose or 2% xylan for 4, 8 or 24 h. The cells were examined using a fluorescence microscope (Olympus BX51) with excitation filters set to 542–582 nm and emission filters set to 604–644 nm. An Olympus 100 oil immersion objective and 10 × oculars were used

To investigate extracellular xylan degradation and the uptake of hydrolysis products such as xylose and oligoxylosides into yeast cells*,* we used a previously constructed fluorescent reporter cassette under the control of the *B. raffinosifermentans XYL1* promoter [12]. The L1-24 strain was transformed with the p*XYL1*-DsRed plasmid (Additional file 2). The recovery of hygromycin-resistant colonies indicates that the genetic engineering tools developed for *B. raffinosifermentans* are functional in *T. vanleenenianus*. A single transformant was cultivated in media containing 2% xylan, 2% xylose or 2% glucose, and fluorescence was monitored after 4, 8 and 24 h of growth. No fluorescence was observed in cells grown in glucose-containing medium (Fig. 6). In contrast, fluorescence was detected after four hours in xylose-containing medium and persisted for up to 24 h. In a xylan-containing medium, fluorescence appeared only after eight hours of growth (Fig. 6). DsRed expression reflects the induction of the *XYL1* promoter in the cells. This induction likely results from the combined action of secreted xylanolytic enzymes and the subsequent uptake of xylan degradation products, such as xylose, as previously described in filamentous fungi [50]. A complementary hypothesis that remains to be tested is that *XYL1* induction may be also triggered by the intracellular degradation of oligoxylosides through enzymes such as Xyn2/Xyl2 (Fig. 4). Further experimental validation is required to confirm this hypothesis and, more broadly, to clarify the functions of the candidate genes identified in this study.

## Discussion

Promising solutions for making biomass recovery processes more efficient, sustainable and competitive lie in exploiting existing microbial diversity and optimising microbial strains. In this perspective, we explored new niches represented by the yeast microbiota of saproxylic insect larvae. We isolated two strains of a species very close to *B. illinoisensis*, which turned out to belong to the *T. vanleenenianus* species described in 2018 [14]. Having detected extracellular xylanase activity [12], we sequenced the entire genome of this strain and identified its hemicellulolytic potential through a transcriptomic approach and a thorough analysis of protein targeting sequences. This study provides the second assembled genome of the genus *Blastobotrys/Trichomonascus*, after *T. ciferrii,* with an expert annotation supported by RNA-seq data [34].

Based on the predicted proteome of strain L1-24, we showed that this strain possesses a complete set of enzymes capable of depolymerising hemicellulosic polysaccharides, such as xylan and xyloglucan. Our study revealed that only the species *B. illinoisensis*, *B. malaysiensis* and *B. mokoenaii* in the *Blastobotrys* genus possess such a gene repertoire*.* These species were isolated from similar environments, i.e., the guts of cetonia larvae or earthworms, cave sediment samples and soil. Such environments may contain rotting or deteriorating wood, which favours the growth of cellulolytic and hemicellulolytic microorganisms. Members of the Trichomonascaceae family are commonly isolated from similar environments. For example, *Sugiyamaella lignohabitans* was isolated from a decaying log and thus exhibits numerous enzymatic activities against plant cell walls [51].

Particular proteins usually absent from Saccharomycotina were found in *T. vanleenenianus,* such as the GH11 endoglucanase Xyn1, which is highly expressed in xylan containing media and possesses a secretion signal. Secreted pectinase (GH28), cellulase (GH5_5) and chitosanase (GH75) are also predicted, enlarging the possibilities of substrate degradation. We also identified proteins with unexpected localization. The GH115 α-glucuronidase, Agu1, known to be secreted in Pezizomycotina species, is predicted to be localized in the cytoplasm and is induced in xylan medium. Similarly, the xylan-induced GH51_1 α-L-arabinosidase is cytoplasmic. The Xyn1 activity produces trixylosides to which glucuronic acid or L-arabinose is attached [52]. These substituted oligoxylosides then enter cells, where intracellular hemicellulolytic enzymes further hydrolyse them. Interestingly, a GH35 enzyme, which is predicted to be cytoplasmic, is also induced in the xylan medium. This is the first report of putative xylanolytic activity in proteins belonging to the GH35 family.

Xylose and oligoxylosides can enter the cells thanks to the xylan-specific expression of different transporters. Comparing the two xylan-induced sugar transporters with known xylose transporters shows that at least one of them has two key motifs important for xylose transport: the first with the sequence GGLXXGYD/N, and the second a five–amino acid sequence rich in aromatic residues (FILYY) [53].

The activity of the intracellular GH5_22 enzyme would complete the saccharification process by acting as either a xylosidase or a xylanase. The induction of GH5_22 enzyme expression in xylan-containing media is consistent with hydrolysis of xylo-oligosaccharides, as reported for CAZymes belonging to this GH5 subfamily. Among the 332 genome-sequenced budding yeasts analysed [10], 126 possess GH5_22 genes, and most *Blastobotrys* species included in our genomic analysis harbor a GH5_22 member likely displaying similar activity. In contrast, in our transcriptomic experiment, the gene encoding the GH5_49 enzyme, also widely distributed among *Blastobotrys* species, was not upregulated in xylan containing medium, suggesting a lack of xylanolytic activity. This observation differs from the endoxylanase activity reported for the *Wickerhamomyces canadensis* GH5_49 protein [46]. Finally, the upregulation of the genes encoding xylose reductase, xylitol dehydrogenase, and xylulose kinase further supports the ability of L1-24 to assimilate xylose.

*T. vanleenenianus* and its three closely related species, have a complete repertoire of xylanolytic enzymes, which is rare for yeast [11, 46]. Furthermore, species in the *Blastobotrys* genus can grow on various sugars, including the single pentose sugars xylose and arabinose, as well as the disaccharides sucrose, cellobiose and xylobiose. These growth characteristics make them particularly well-suited to the valorisation of plant biomass. Well-known chassis such as *Y. lipolytica* or *S. cerevisiae* are unable to utilize pentoses naturally. However, the inability of *T. vanleenenianus* to grow at temperatures above 30 °C, coupled with its hyphal morphology, poses challenges for its industrial application. A contrario, *B. raffinosifermentans* assimilates xylose and is thermotolerant. Furthermore, *B. raffinosifermentans* naturally produces a glucuronidase of the GH67 family and two xylosidases, all of which are secreted. This yeast could be used as a chassis to express some of the genes involved in hemicellulose degradation. In this regard, *T. vanleenenianus* can be considered a reservoir of enzymes of interest. One of the most interesting of these is the endoxylanase Xyn1. Overexpressing it in *B. raffinosifermentans* using our genetic tools [12] will complete the set of hemicellulolytic enzymes and enable us to produce high-value products from plant biomass.

## Conclusion

In conclusion, this study highlights the remarkable enzymatic potential of *Trichomonascus vanleenenianus* (strain L1-24) and its most closely related *Blastobotrys* species. Through genome and transcriptomic analyses, we revealed a comprehensive and functionally diverse hemicellulolytic system that is capable of saccharifying complex polysaccharides, such as xylan. The presence of a large diversity of extracellular and cytoplasmic enzymes — some of which are typically absent in other Saccharomycotina yeasts — illustrates the adaptation of *T. vanleenenianus* to lignocellulose-rich environments and its metabolic versatility with regard to pentose assimilation. Furthermore, the coordinated expression of transporters and enzymes that break down hemicellulose and enable the utilization of pentoses promotes both an efficient saccharification process and the further assimilation of released xylan-derived sugars. Despite its industrial limitations, such as temperature sensitivity and filamentous growth, *T. vanleenenianus* represents a valuable reservoir of enzymes with significant potential for biomass valorization. The transfer of key genes, such as *XYN1*, into thermotolerant yeasts like *B. raffinosifermentans* could pave the way for the development of robust microbial platforms optimized for plant biomass conversion. Overall, our findings illustrate that exploring yeast biodiversity contributes to enlarge the range of enzymes available for sustainable bio-based production and highlight the potential of novel yeasts in white biotechnology.

## Supplementary Information

Additional file 1: Genomes available as of November 11, 2025.

Additional file 2: Construction of the DsRed reporter plasmid.

Additional file 3: Genetic features annotated in the genome of L1-24.

Additional file 4: ANI values computed between 5 strains of *Blastobotrys *and *Trichosmoascus *species.

Additional file 5: Characteristics of the 2,539 spliceosomal introns.

Additional file 6: MAT locus of *T. vanleenenianus* in comparison to those of other species in the genus and *Y. lipolytica.*

Additional file 7: Genes involved in mating in L1-24 and other Saccharomycotina yeasts

Additional file 8: Secreted proteins. A. List of the 45 predicted GPI-anchored proteins. B. List of the 157 predicted extracellular proteins.

Additional file 9: List of the 127 T. vanleenenianus CAZymes of the families AA, CBM, CE and GH

Additional file 10: Alignment of the ten secretory aspartic proteinases from *T. vanleenenianus *L1-24.

Additional file 11: Lipases from *T. vanleenenianus*, *B. raffinosifermentans *and *T. ciferrii.*

Additional file 12: Alignment and phylogenetic tree of the GH5_5 proteins from *T. vanleenenianus*,* B. illinoisensis, B. malaysiensis, B. mokoenaii *and *B. proliferans* as an outgroup.

Additional file 13: Diversity of GH11 proteins.

Additional file 14: List of the 194 genes differentially expressed between glucose and xylan. **A**. List of the 102 genes up-regulated in xylan. **B**. List of the 92 genes down-regulated in xylan.

## Data Availability

The genome and transcriptome sequencing data have been deposited in the European Nucleotide Archive at EMBL-EBI under the accession number PRJEB90485, the assembly accession number is GCA_965637985. Strains L1-24 (= CLIB 3782) and L2-36 (= CLIB 3783) have been deposited in the CIRM-Levures yeast collection (https://cirm-levures.bio-aware.com).
