# Supplementary material for: Genomic and transcriptomic insights into Trichomonascus vanleenenianus, a xylan-degrading yeast isolated from saproxylic insect larvae
Source: BMC Genomics. 2026 Mar 21;27:422. doi: 10.1186/s12864-026-12750-7 (PMC13130702; doi:10.1186/s12864-026-12750-7)
Supplement: Supplementary file 2 — Additional file 2: Construction of the DsRed reporter plasmid. [file 12864_2026_12750_MOESM2_ESM.pdf]

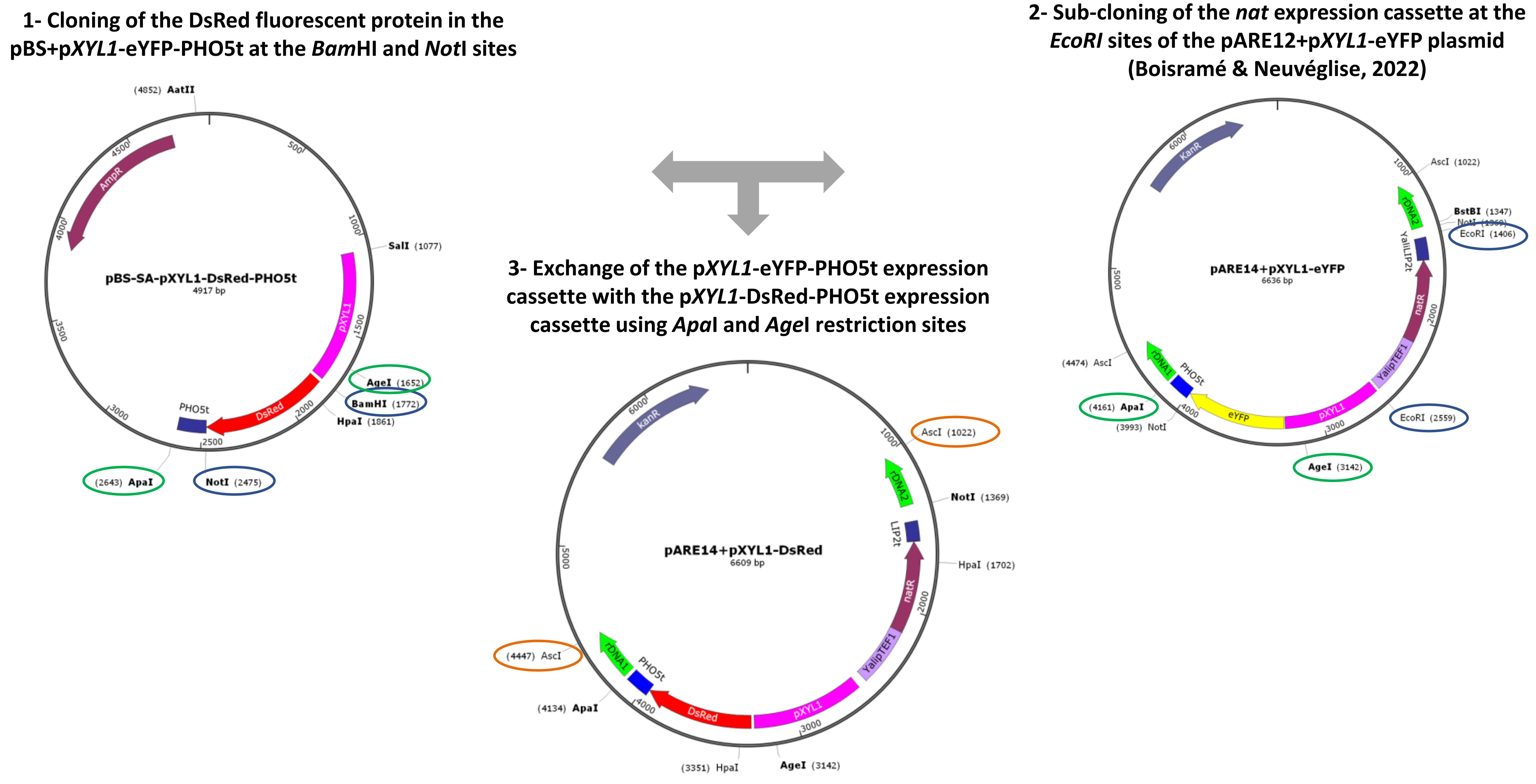

Additional file 2: Construction of the DsRed reporter plasmid.
