# Supplementary material for: Genomic and transcriptomic insights into Trichomonascus vanleenenianus, a xylan-degrading yeast isolated from saproxylic insect larvae
Source: BMC Genomics. 2026 Mar 21;27:422. doi: 10.1186/s12864-026-12750-7 (PMC13130702; doi:10.1186/s12864-026-12750-7)
Supplement: Supplementary file 5 — Additional file 5: Characteristics of the 2,539 spliceosomal introns. [file 12864_2026_12750_MOESM5_ESM.pdf]

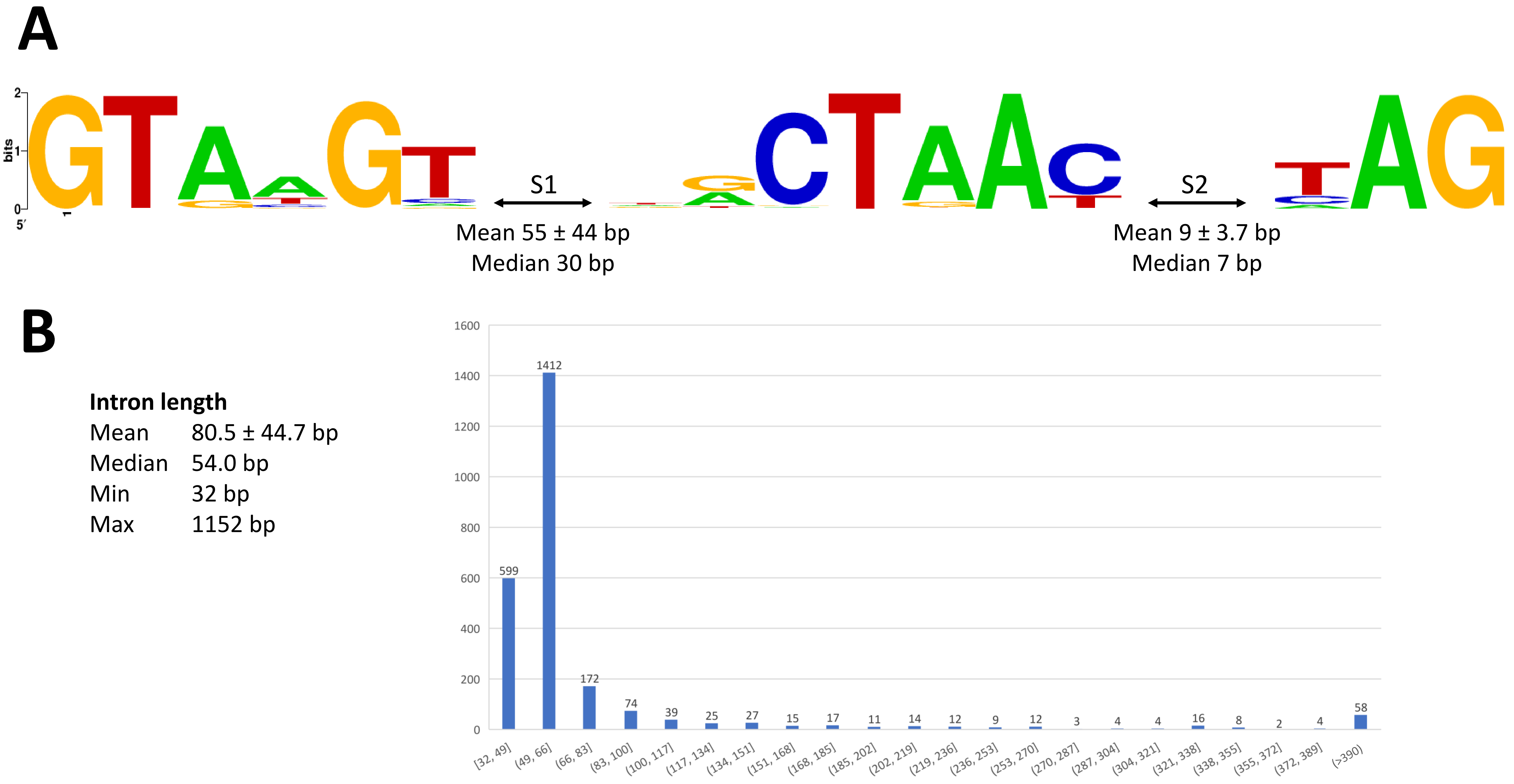

Additional file 5: Characteristics of the 2,539 spliceosomal introns. **A**. Patterns of the 5′ splice site, branch point and 3′ splice site, along with the mean and the median of the S1 and S2 distances. **B**. Distribution of intron length.
