# Supplementary material for: Genomic and transcriptomic insights into Trichomonascus vanleenenianus, a xylan-degrading yeast isolated from saproxylic insect larvae
Source: BMC Genomics. 2026 Mar 21;27:422. doi: 10.1186/s12864-026-12750-7 (PMC13130702; doi:10.1186/s12864-026-12750-7)
Supplement: Supplementary file 6 — Additional file 6: MAT locus of T. vanleenenianus in comparison to those of other species in the genus and Y. lipolytica. [file 12864_2026_12750_MOESM6_ESM.pdf]

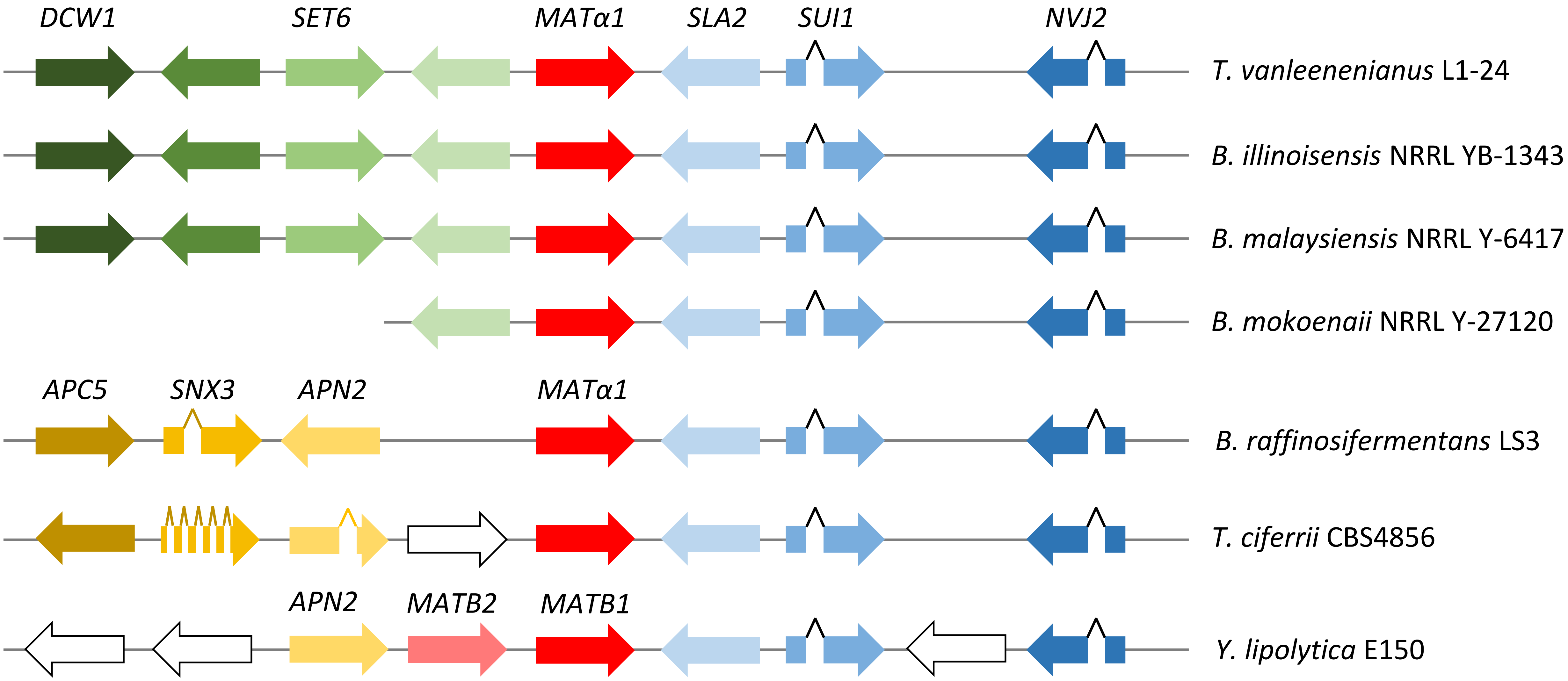

Additional file 6: *MAT* locus of *T. vanleenenianus* in comparison to those of other species in the genus and *Y. lipolytica.* Conserved genes are represented by arrows with the same colour. MATα1 (MATB1 in *Y. lipolytica*) is shown in red. Genes without homologues in the MAT locus are represented by white arrows. Spliceosomal introns are represented by an interruption in the CDS, indicated by a pointed hat consisting of two lines.
