# Supplementary material for: Genomic and transcriptomic insights into Trichomonascus vanleenenianus, a xylan-degrading yeast isolated from saproxylic insect larvae
Source: BMC Genomics. 2026 Mar 21;27:422. doi: 10.1186/s12864-026-12750-7 (PMC13130702; doi:10.1186/s12864-026-12750-7)
Supplement: Supplementary file 10 — Additional file 10: Alignment of the ten secretory aspartic proteinases from T. vanleenenianus L1-24. [file 12864_2026_12750_MOESM10_ESM.pdf]

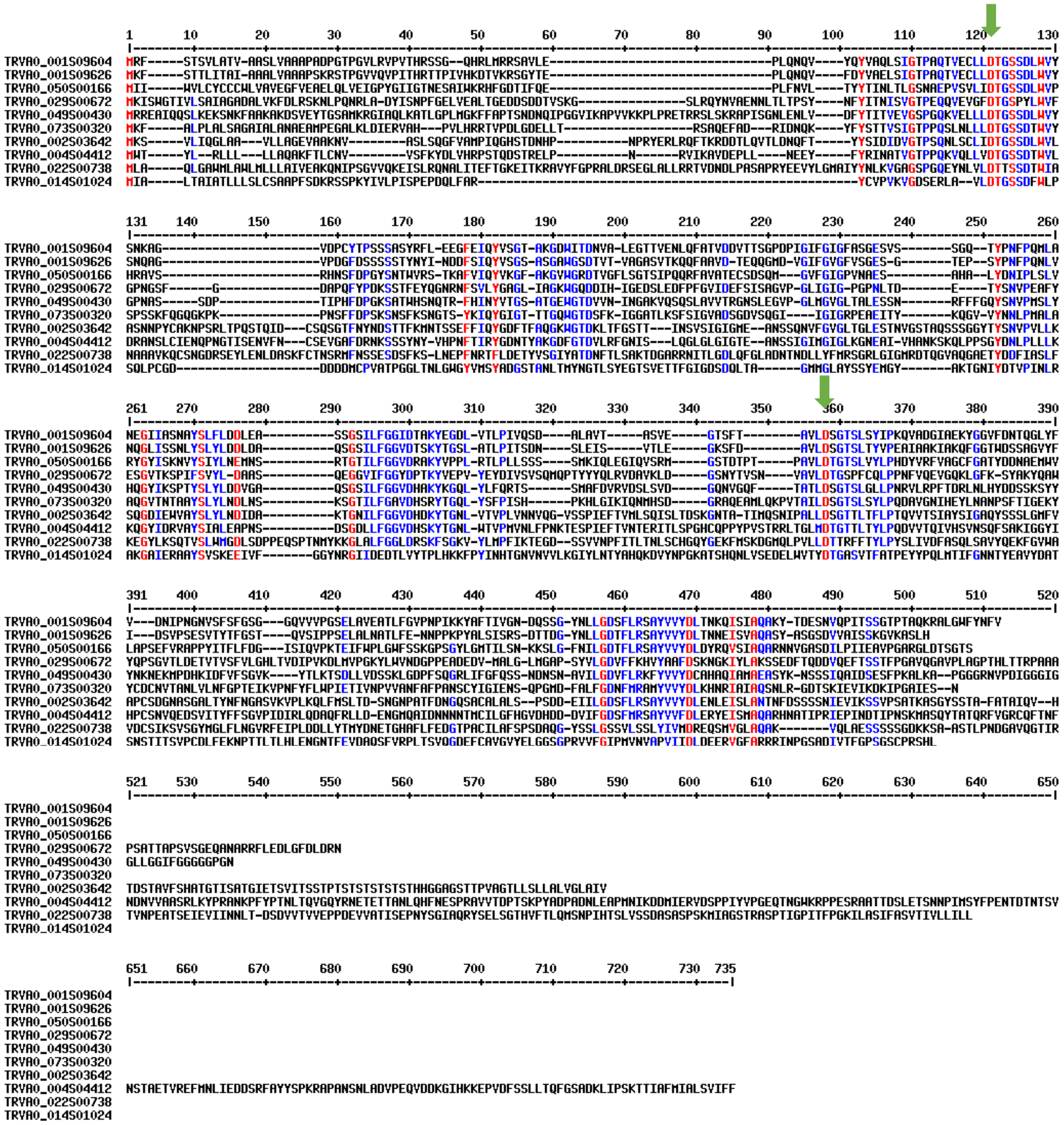

Additional file 10: Alignment of the ten secretory aspartic proteinases from *T. vanleenenianus* L1-24.

Alignment was performed with the Multalin server (http://multalin.toulouse.inra.fr/multalin/). The two conserved catalytic aspartic acids are shown by green arrows. The first 200 amino acids from TRVA0_049S00430 were discarded to optimize the alignment.
