# Supplementary material for: Genomic and transcriptomic insights into Trichomonascus vanleenenianus, a xylan-degrading yeast isolated from saproxylic insect larvae
Source: BMC Genomics. 2026 Mar 21;27:422. doi: 10.1186/s12864-026-12750-7 (PMC13130702; doi:10.1186/s12864-026-12750-7)
Supplement: Supplementary file 11 — Additional file 11: Lipases from T. vanleenenianus, B. raffinosifermentans and T. ciferrii. [file 12864_2026_12750_MOESM11_ESM.pdf]

A

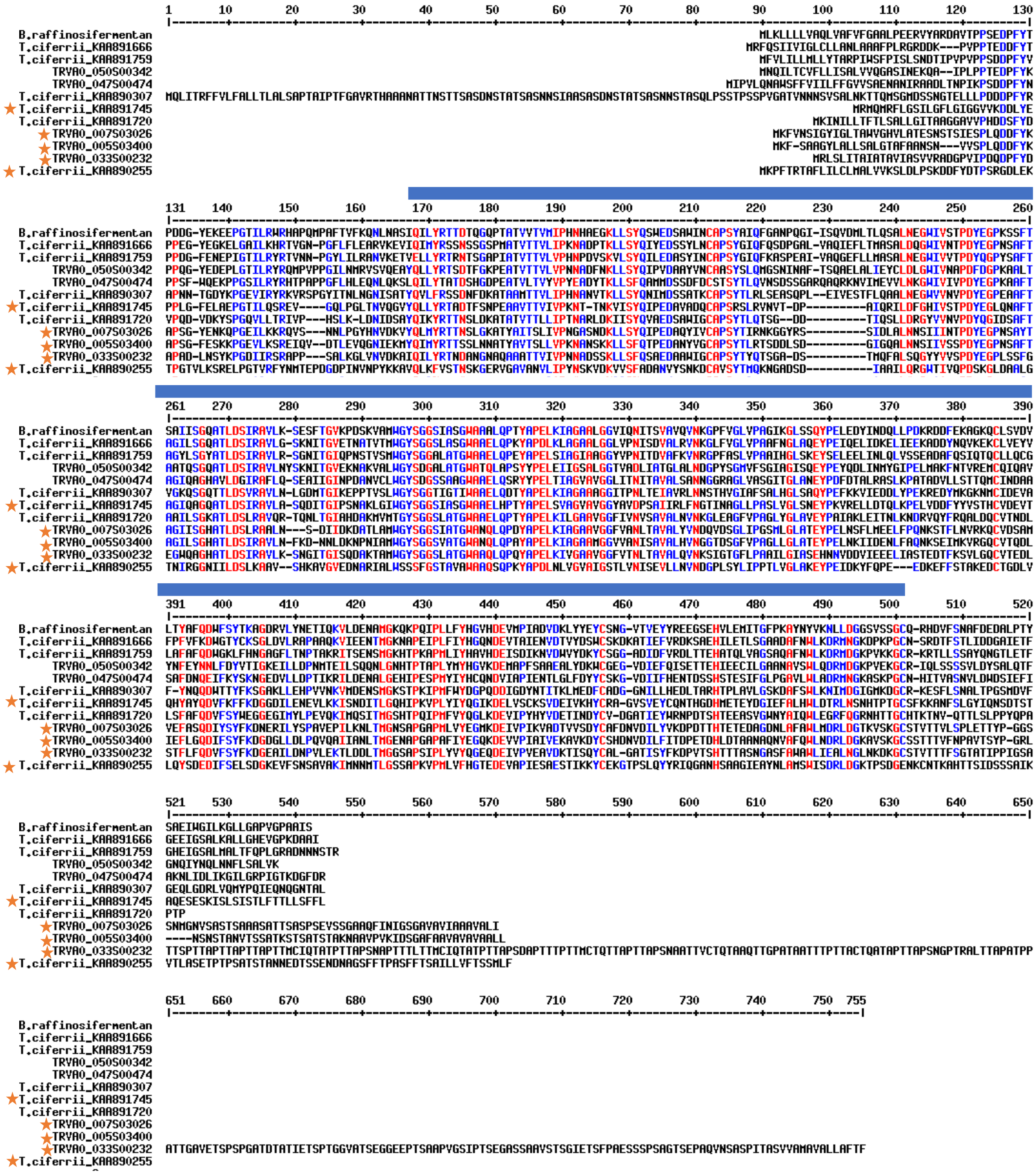

B.raffinosifermentan
T.ciferrii_KAA891666
T.ciferrii_KAA891759
TRVA0_050S00342
TRVA0_047S00474
T.ciferrii_KAA890307
T.ciferrii_KAA891745
T.ciferrii_KAA891720
TRVA0_007S03026
TRVA0_005S03400
TRVA0_033S00232
T.ciferrii_KAA890255

B

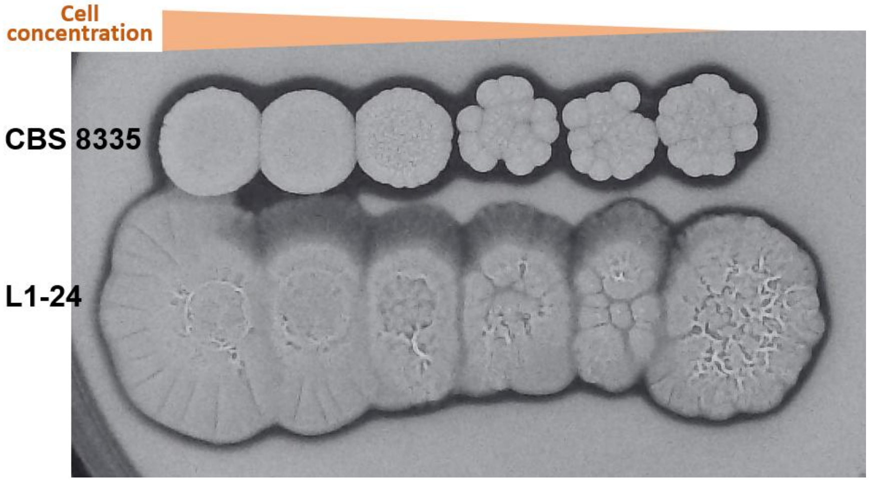

Additional file 11: Lipases from *T. vanleenenianus, B. raffinosifermentans* and *T. ciferrii*.

A. Alignment was performed using the Multalin server (http://multalin.toulouse.inra.fr/multalin/). The five lipases from *T. vanleenenianus* were aligned with one lipase from *B. raffinosifermentans* LS3 (accession number: Q5GMI6) and six lipases from *T. ciferrii* (accession numbers: KAA8916660.1, KAA8917591.1, KAA8903072.1, KAA8917458.1, KAA8917206.1, KAA8902559.1). The pfam03583 conserved domain is highlighted in blue. Orange stars indicate GPI-anchored lipases. **B**. Growth of L1-24 and CBS 8335 (*B. raffinosifermentans*) on YNB tributyrin after incubation for one week at 30 °C. Drops of 5 µL are diluted by a factor of 10 from left to right.
