# Supplementary material for: Genomic and transcriptomic insights into Trichomonascus vanleenenianus, a xylan-degrading yeast isolated from saproxylic insect larvae
Source: BMC Genomics. 2026 Mar 21;27:422. doi: 10.1186/s12864-026-12750-7 (PMC13130702; doi:10.1186/s12864-026-12750-7)
Supplement: Supplementary file 12 — Additional file 12: Alignment and phylogenetic tree of the GH5_5 proteins from T. vanleenenianus, B. illinoisensis, B. malaysiensis, B. mokoenaii and B. proliferans as an outgroup. [file 12864_2026_12750_MOESM12_ESM.pdf]

A

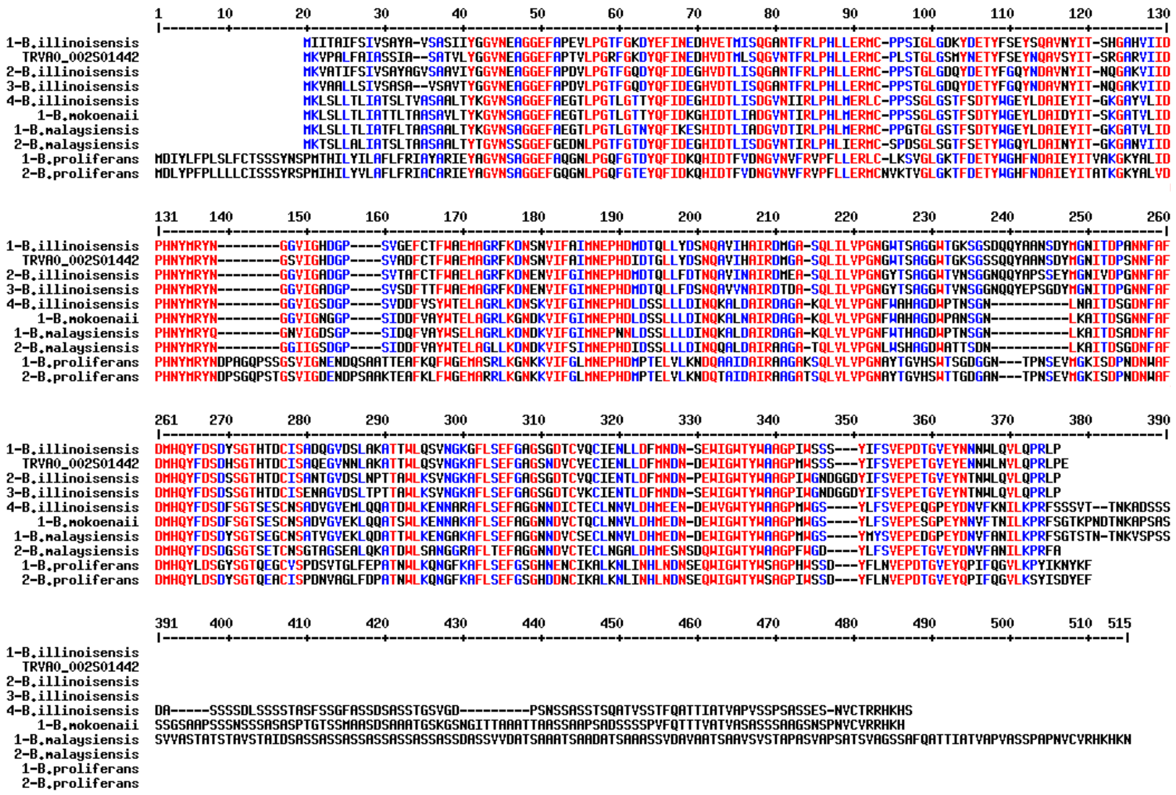

B

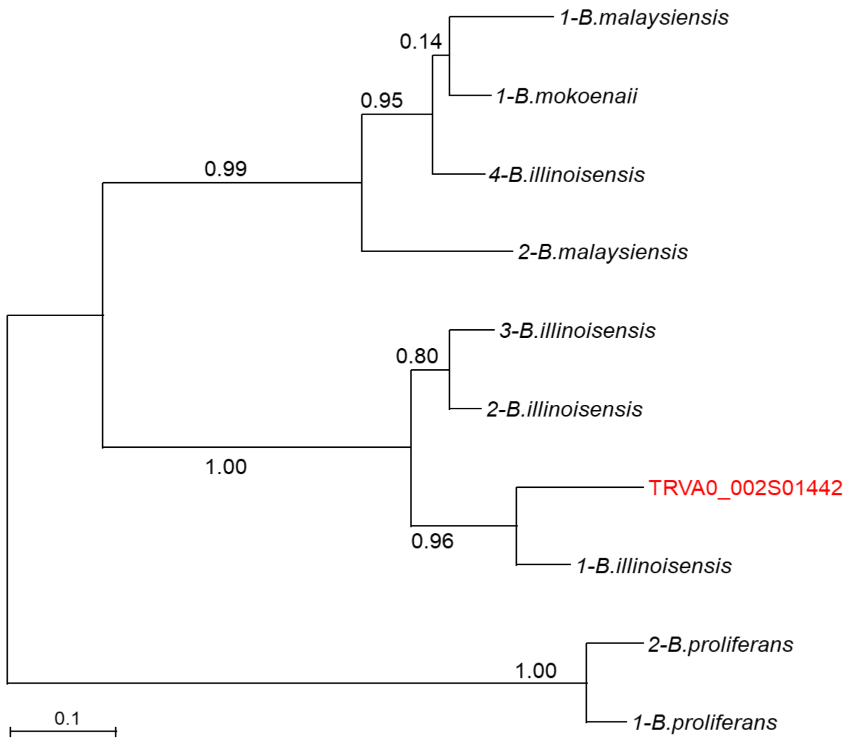

Additional file 12: Alignment and phylogenetic tree of the GH5_5 proteins from *T. vanleenenianus*, *B. illinoisensis*, *B. malaysiensis*, *B. mokoenaii* and *B. proliferans* as an outgroup.

A. Alignment was performed using the Multalin server (http://multalin.toulouse.inra.fr/multalin/). The *T. vanleenenianus* GH5_5 protein, which is encoded by the TRVA0_002S01442 gene, was aligned with four *B. illinoisensis,* two *B. malaysiensis* and one *B. mokoenaii* GH5_5 proteins. *B. proliferans* GH5_5 proteins were used as outgroups. **B.** A phylogenetic tree was computed using PhyML v3.0 with the LG model and four rate classes, based on 327 sites. The approximate likelihood ratio test (aLRT) approach was used to estimate branch support. The protein of *T. vanleenenianus* L1-24 is shown in red.
