# Supplementary material for: Genomic and transcriptomic insights into Trichomonascus vanleenenianus, a xylan-degrading yeast isolated from saproxylic insect larvae
Source: BMC Genomics. 2026 Mar 21;27:422. doi: 10.1186/s12864-026-12750-7 (PMC13130702; doi:10.1186/s12864-026-12750-7)
Supplement: Supplementary file 13 — Additional file 13: Diversity of GH11 proteins. [file 12864_2026_12750_MOESM13_ESM.pdf]

A

		1	2	3	4	5	6	7	8	9	10	11	12	13	14	15
Trichomonascus vanleenenianus	1	100%	77%	71%	72%	64%	65%	64%	64%	66%	63%	66%	63%	40%	31%	34%
Blastobotrys illinoisensis	2		100%	85%	88%	62%	62%	61%	62%	65%	62%	63%	62%	40%	32%	32%
Blastobotrys malaysiensis	3			100%	85%	61%	62%	62%	63%	64%	63%	63%	62%	40%	32%	36%
Blastobotrys mokoenaii	4				100%	62%	65%	63%	62%	65%	63%	66%	63%	41%	33%	34%
Aspergillus niger	5					100%	67%	69%	75%	72%	96%	70%	94%	43%	33%	36%
Aspergillus fumigatus	6						100%	78%	69%	72%	67%	92%	67%	40%	35%	37%
Aspergillus nomiae	7							100%	70%	70%	70%	79%	69%	40%	33%	38%
Aspergillus wentii	8								100%	68%	77%	72%	75%	44%	35%	38%
Aspergillus nidulans	9									100%	70%	71%	70%	43%	35%	36%
Aspergillus piperis	10										100%	70%	97%	44%	34%	36%
Aspergillus turcosus	11											100%	69%	40%	34%	38%
Aspergillus luchuensis	12												100%	44%	34%	36%
Aureobasidium pulullans	13													100%	47%	51%
Cryptococcus sp.	14														100%	46%
Pseudozyma antactica	15															100%

30%
40%
50%
60%
70%
80%
90%
100%

B

		1	2	3	4	5	6	7	8	9	10	11	12	13	14	15
Trichomonascus vanleenenianus	1	100%	84%	81%	83%	75%	76%	74%	74%	78%	75%	75%	76%	54%	49%	52%
Blastobotrys illinoisensis	2		100%	91%	94%	71%	72%	72%	70%	75%	71%	71%	72%	50%	46%	49%
Blastobotrys malaysiensis	3			100%	90%	71%	70%	71%	69%	75%	71%	71%	71%	50%	46%	51%
Blastobotrys mokoenaii	4				100%	72%	74%	75%	71%	78%	73%	74%	72%	51%	46%	50%
Aspergillus niger	5					100%	76%	80%	84%	81%	99%	78%	98%	55%	48%	49%
Aspergillus fumigatus	6						100%	86%	78%	79%	77%	96%	76%	53%	50%	54%
Aspergillus nomiae	7							100%	80%	81%	80%	87%	80%	57%	50%	55%
Aspergillus wentii	8								100%	81%	84%	80%	83%	58%	50%	52%
Aspergillus nidulans	9									100%	81%	79%	81%	59%	50%	51%
Aspergillus piperis	10										100%	78%	98%	56%	48%	50%
Aspergillus turcosus	11											100%	77%	52%	49%	55%
Aspergillus luchuensis	12												100%	55%	48%	50%
Aureobasidium pulullans	13													100%	65%	64%
Cryptococcus sp.	14														100%	63%
Pseudozyma antactica	15															100%

40%
50%
60%
70%
80%
90%
100%

Additional file 13: Diversity of GH11 proteins.
Matrix showing the identity (A) and similarity (B) of GH11 proteins. The xylanases most closely related to the *Blastobotrys* and *Trichomonascus* species are found in the *Aspergillus* species.
